# Chimpanzee-Specific Endogenous Retrovirus Generates Genomic Variations in the Chimpanzee Genome

**DOI:** 10.1371/journal.pone.0101195

**Published:** 2014-07-02

**Authors:** Seyoung Mun, Jungnam Lee, Yun-Ji Kim, Heui-Soo Kim, Kyudong Han

**Affiliations:** 1 Department of Nanobiomedical Science, Dankook University, Cheonan, Republic of Korea; 2 BK21 PLUS NBM Global Research Center for Regenerative Medicine, Dankook University, Cheonan, Republic of Korea; 3 DKU-Theragen institute for NGS analysis (DTiNa), Cheonan, Republic of Korea; 4 Departments of Periodontology & Oral Biology, College of Dentistry, University of Florida, Gainesville, Florida, United States of America; 5 Department of Biological Sciences, College of Natural Sciences, Pusan National University, Busan, Republic of Korea; Estonian Biocentre, Estonia

## Abstract

Endogenous retroviruses (ERVs), eukaryotic transposable elements, exist as proviruses in vertebrates including primates and contribute to genomic changes during the evolution of their host genomes. Many studies about ERVs have focused on the elements residing in the human genome but only a few studies have focused on the elements which exist in non-human primate genomes. In this study, we identified 256 chimpanzee-specific endogenous retrovirus copies (PtERVs: *Pan troglodyte* endogenous retroviruses) from the chimpanzee reference genome sequence through comparative genomics. Among the chimpanzee-specific ERV copies, 121 were full-length chimpanzee-specific ERV elements while 110 were chimpanzee-specific solitary LTR copies. In addition, we found eight potential retrotransposition-competent full-length chimpanzee-specific ERV copies containing an intact *env* gene, and two of them were polymorphic in chimpanzee individuals. Through computational analysis and manual inspection, we found that some of the chimpanzee-specific ERVs have propagated via non-classical PtERV insertion (NCPI), and at least one of the PtERVs may have played a role in creating an alternative transcript of a chimpanzee gene. Based on our findings in this study, we state that the chimpanzee-specific ERV element is one of the sources of chimpanzee genomic variations, some of which might be related to the alternative transcripts in the chimpanzee population.

## Introduction

A *Pan troglodytes* endogenous retrovirus (PtERV) is a type of abundant long terminal repeat (LTR) retrotransposons in vertebrate genomes. PtERVs are categorized into three major classes (class I: the gammaretroviruses, class II: the betaretroviruses, and class III: the spumaviruses). They are further divided into 42 families including two chimpanzee-specific ERV families [1]. While the majority of PtERV families are shared in human and Asian ape genomes, CERV1 and CERV2 families have been shown only in the African great ape and Old World monkey genomes. It was suggested that the two families originated from an ancient viral infection within a germ-line cell after the divergence of chimpanzees and humans [2]. Since then, their copy numbers have been increased by vertical transmission and retroviral infections [3].

Similar to other ERVs, PtERVs contain four internal genes: group-specific antigen (*gag*), protease gene (*pro*), RNA-dependent DNA polymerase gene (*pol*), and envelope gene (*env*), which are flanked by LTRs. Especially, the *env* gene encodes for the viral protein called envelop glycoprotein (Env), which mediates virus entry into a host cell via receptor-mediated endocytosis [4]–[6]. Interestingly, some PtERVs retain the structural coding gene (an intact *env* gene), indicating that they are potentially infectious in the chimpanzee genome. However, other typical ERVs are generally deficient in the *env* gene because they have been rapidly decayed by either accumulated point mutations [7] and/or selection on the host genome such as DNA methylation [8] or the recombination between LTRs [9].

There is an overall genomic difference of ∼4% between humans and chimpanzees, and the mechanisms causing the genomic difference include single-nucleotide divergence and insertion/deletion (INDEL) events ranging from 1 to 10,000 bp [10], [11]. PtERVs are among the factors causing INDELs in human and chimpanzee genomes and are responsible for ∼7% of the 4% genome-wide difference between the two species. It has been known that ERVs play a role in creating structural variations in the genome of their hosts through various mechanisms including *de novo* insertion, homologous recombination between LTRs, and frameshift mutations in the host genes [12], [13]. In addition, retroviral insertion can generate gene transcript variations by altering the gene transcription. The element could delete exon(s), create novel exon(s) or serve as a splice donor or acceptor. In fact, ERVs are considered to be significant factors in the production of alternative transcripts [14]–[16].

Here, we investigated chimpanzee-specific ERV copies, which were integrated in the chimpanzee genome after the divergence of humans and chimpanzees. Through combined computational data mining and manual inspection, we identified a total of 256 chimpanzee-specific ERV elements in the chimpanzee reference genome (panTro3). Among them, we examined 121 full-length chimpanzee-specific ERV copies and 110 chimpanzee-specific solitary LTR copies, focusing on their structures, chromosomal distributions and genomic contexts. Then, we further characterized them through the pairwise distance test, and estimated their polymorphism levels in chimpanzees.

## Results and Discussion

### Identification and characterization of chimpanzee-specific ERVs

To identify chimpanzee-specific ERV insertions, we computationally identified 693 PtERV candidates from the chimpanzee reference genome sequence based on RepeatMasker annotation [17]-[19]. Then, we manually inspected the candidates, as described in the materials and methods section. The manual inspection showed that many of the PtERV candidates were actually shared between the chimpanzee and other non-chimpanzee primate genomes, so the false positives were eliminated from our data. The result is summarized in Table 1. Our data contained 121 full-length chimpanzee-specific ERV copies, and among them were 14 new full-length elements which have not been identified in previous studies [1], [2].

**Table 1:** Summary of chimpanzee-specific ERV loci.

Classification	No. of loci
*Computationally predicted PtERV loci*	693
*Number of chimpanzee-specific ERV insertion events*	256
Full-length chimpanzee-specific ERV insertions	121
Chimpanzee-specific solitary LTR insertions	110
Truncated PtERV insertions	22
Unknown types of PtERV insertions	3

Of the 42 PtERV families established by previous studies, only CERV1 and CERV2 are chimpanzee-specific [1], [2]. PtERVs have been grouped based on the sequence of their LTRs. Thus, we performed multiple sequence alignments of full-length chimpanzee-specific ERV copies based on their LTR sequences to determine which PtERV family each element belonged to. As we expected, most of the elements belonged to the two families of CERV1 and CERV2. However, upon closer examination, we found significant sequence variations within each of the families: the CERV1 family can be divided into two subfamilies (CERV1 and PtERV-1c) and the CERV2 family also can be divided into four subfamilies (CERV2, PtERV-2a, PtERV-2b, and PtERV-2c) according to the INDEL pattern (Figure S1). In summary, our data contained six different PtERV subfamilies, each of which belonged to either the CERV1 or the CERV2 family. Among the 256 chimpanzee-specific ERV copies, 207 elements belonged to the CERV1 family: 83 to the CERV1 subfamily and 124 to the PtERV-1c subfamily. Only 24 elements belonged to the CERV2 family: three to PtERV-2a, four to PtERV-2b, sixteen to PtERV-2c, and one to CERV2 subfamily. We were not able to determine the subfamily of the remaining 25 elements because they were truncated or unknown type. In addition, we constructed a phylogenetic relationship among the different chimpanzee-specific ERV subfamilies based on their 5′ and 3′ LTR consensus sequences (Figure S2). As a result, the two LTRs of an ERV copy tend to have a high sequence identity to one another.

We identified 110 chimpanzee-specific solitary LTR copies from the chimpanzee genome. Among them, 87 LTRs were previously identified elements [2] and 23 were new elements found only in this study. Previous studies about HERV-Ks reported that most of them exist as a solitary LTR in the human genome. One of the studies found 128 human-specific solitary LTRs, which are more than the number of human-specific full-length HERV-Ks in the human genome [20]. Unlike HERV-K, the number of chimpanzee-specific solitary LTR copies is similar to that of the full-length chimpanzee-specific ERV copies. This finding indicates that, since the divergence of humans and chimpanzees, LTR-LTR homologous recombination between PtERVs has occurred at a lower frequency than that between HERV-Ks. We estimated the substitution rate of LTRs from HERV-K and each family of PtERVs. The substitution rate of HERV-K LTR was 0.009, and those of CERV1 and CERV2 were 0.040 and 0.011, respectively. As such, this result suggests that compared to HERV-K LTRs, the two LTRs of a PtERV element share slightly lower sequence similarity due to the accumulation of nucleotide substitutions on them. We suggest that the sequence identity between the two LTRs of an ERV element is responsible for the difference between the rates of solitary HERV-K LTRs and PtERV LTRs. In addition, the percent of solitary LTR copies from CERV1 family (45.9%) is lower than that from CERV2 family (62.5%) because LTR sequences of CERV2 family are less diversified within each subfamily.

**Figure 1:**
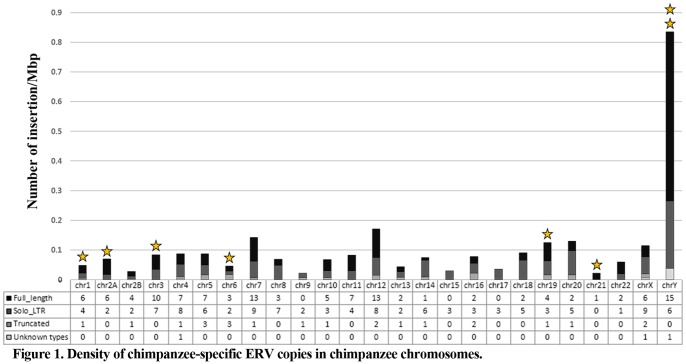
Density of chimpanzee-specific ERV copies in chimpanzee chromosomes. Each bar indicates the density of chimpanzee-specific ERV copies (number of insertion/Mbp of sequences) on each chimpanzee chromosome. The distribution bar is composed of four different colors, each of which represents one of four different PtERV categories: full-length chimpanzee-specific ERV, chimpanzee-specific solitary LTR, truncated PtERV, and unknown types of PtERV. Yellow stars indicate the presence of potential rcPtERVs in the respective chromosome.

We examined the chromosomal distribution of full-length chimpanzee-specific ERV elements in the chimpanzee genome. As shown in Figure 1, full-length chimpanzee-specific ERV copies exist in relatively high frequencies on chromosomes 7, 12, and Y. Especially, chromosome Y contains 15 full-length chimpanzee-specific ERV loci. Based on the density of insertions (number of insertion/Mbp of sequences), we found preferential accumulation of chimpanzee-specific ERV copies to Y chromosome (Figure 1) that is congruent with previous studies of human and primate Y chromosomes [17], [21]. Recombination is one of the mechanisms removing ERV copies from the host genome. As such, we believe that chimpanzee-specific ERV copies were able to accumulate on Y chromosome due to the low recombination frequency of the chromosome.

**Figure 2:**
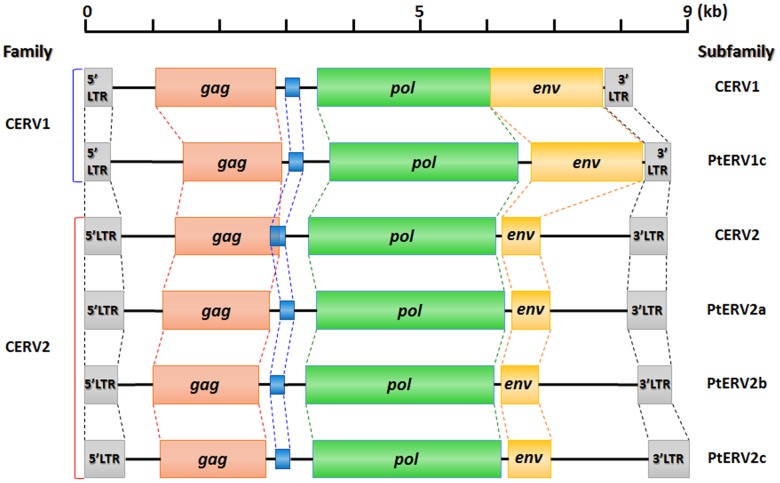
Structure of chimpanzee-specific ERVs. This figure depicts the structure of six different chimpanzee-specific ERV subfamilies, deposited in the CENSOR database. Each colored box indicates a gene; the red, blue, green, and orange boxes indicate *gag*, *pro*, *pol*, and *env* gene, respectively. The grey box indicates LTR sequence.

Further we characterized chimpanzee-specific ERV insertions, focusing on their size. For the size analysis, we excluded 22 truncated chimpanzee-specific ERV copies from our data. Initially, we retrieved six different consensus sequences of full-length chimpanzee-specific ERV copies; each consensus sequence corresponds to one PtERV subfamily. The sizes of the six consensus sequences range from 8.6 to 9 kb. Then, we performed sequence alignments of the consensus sequence and the full-length chimpanzee-specific ERV copies, and found that the size of a chimpanzee-specific ERV was variable even within a subfamily, which resulted from independent INDELs of individual members of each subfamily. We compared the consensus sequences from 6 different PtERV subfamilies, focusing on their internal genes. The subfamilies did not show a significant difference in *gag*, *pro*, and *pol* genes. However, there was a remarkable sequence variability between *env* genes from different families; *env* gene of CERV2 family is shorter than that of CERV1 family, although the *env* domain of CERV2 family retains complete surface (SU) and transmembrane (TM) motifs (Figure 2). The sizes of chimpanzee-specific ERV copies were also variable; the ERV copies of CERV1 family ranged in size from 6.4 to 9.9 kb, and those of CERV2 family ranged in size from 8.4 and 9.0 kb. We also compared 6 different PtERV subfamilies, focusing on their LTR sequences. The result showed that each subfamily has a unique sequence feature on the LTR region and PtERV-2c subfamily has the longest LTR, 603 bp (Table 2). Furthermore, we compared the LTR sequences between the PtERV-1c and the CERV1 subfamily members, and between four different CERV2 subfamily members in Figure S1. The results indicate that LTR sequences of each subfamily are diversified including INDELs. To examine whether the chimpanzee-specific ERV copies have a target site preference for integration, we examined target site duplications (TSDs) of each chimpanzee-specific ERV. Target site sequences are described in Table S1 and S2. The comparison of the TSD sequences revealed no specific target sequence for chimpanzee-specific ERV integration. Based on this result, we suggest that chimpanzee-specific ERVs are randomly integrated into the chimpanzee genome.

**Table 2:** Analysis of chimpanzee-specific ERV LTRs.

Classification		Copy number	No. of Full-length insertions	No. of Solitary LTRs	Average length of each LTR subfamily
CERV1 family	CERV1 subfamily	83	51	32	409
	PtERV-1c subfamily	124	61	63	379
CERV2 family	CERV2 subfamily	1	1	-	544
	PtERV-2a subfamily	3	2	1	586
	PtERV-2b subfamily	4	1	3	491
	PtERV-2c subfamily	16	5	11	603

### Polymorphism level of chimpanzee-specific ERVs

Through locus-specific PCR amplification, we examined the polymorphism level of chimpanzee-specific ERV copies within a common chimpanzee population panel composed of 12 related individuals from Primate Research Institute, Kyoto University. For the examination, we randomly chose 20 chimpanzee-specific solitary LTR loci and 28 full-length chimpanzee-specific ERV loci containing 20% of CERV1 family and 20% of CERV2 family using a statistic program (Doug's Random Sampling Applet web site) [22], and 4 additional retrotransposition-competent PtERV (rcPtERV) loci. For the full-length chimpanzee-specific ERV copies, we used an internal forward or reverse primer to amplify the respective loci. Two primer combinations were possible: One combination is the forward primer from the flanking sequence and the internal reverse primer, and the other combination is the internal forward primer and the reverse primer from the flanking sequence. We designed the internal forward and internal reverse primers from the *env* and *gag* regions, respectively. Using these primers, we successfully amplified the 52 loci through PCR with DNA templates from the 12 chimpanzee individuals (Figure S3). Similar to HERVs (i.e., LTR-retrotransposons), PtERVs exhibit three different forms (i.e., trimorphism): absence of the PtERV element, presence of the PtERV element, and presence of a solitary LTR, which can result from the homologous recombination between two LTRs [1], [12], [23], [24]. The 52 chimpanzee-specific PtERV loci were subjected to a polymorphic test. The result showed that 20.9% (9/43) of CERV1 copies and 22.2% (2/9) of CERV2 copies are polymorphic in the chimpanzee individuals (Table S3, S4, and S5). To examine whether any of the solitary LTR loci exist in trimorphism, we performed PCR of the 20 solitary LTR loci using the PtERV conserved internal primer and a flanking primer. None of the solo-LTR loci were trimorphic in the chimpanzee individuals but one of them (PtERV#216) displayed polymorphism: solitary LTR and presence of the ERV copy. The polymorphism rate of CERV2 family is slightly higher than that of CERV1 family, which supports that CERV2 family is relatively younger than CERV1 family. When compared with human-specific HERV-K, chimpanzee-specific ERV copies were by far less polymorphic than human-specific HERV-K (∼48%) [12]. However, the polymorphism rate of chimpanzee-specific ERV copies is possibly underestimated because only 12 related chimpanzee individuals were used for the test.

### Identification of structurally intact chimpanzee-specific ERVs

**Figure 3:**
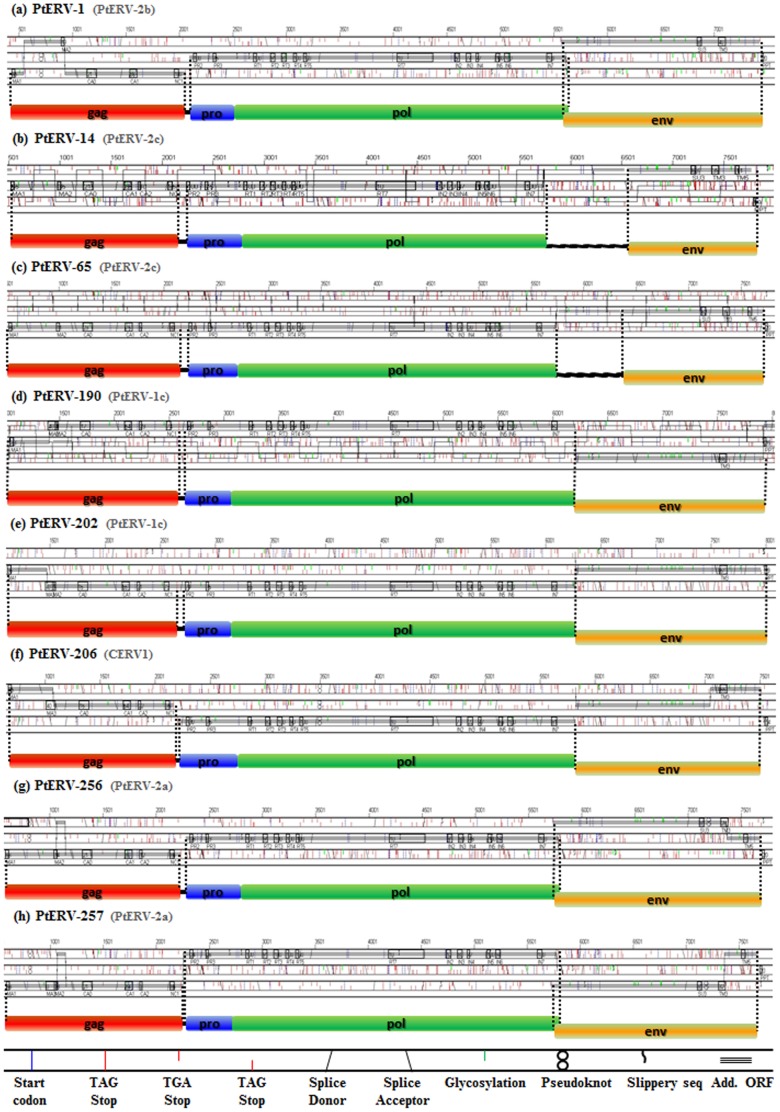
Structural analysis of potential rcPtERVs. Using the RetroTector 10 program, the eight potential rcPtERVs were fragmented into four parts: *gag* (internal structural proteins, motifs named CA and NC), *pro* (protease, motifs named PR), *pol* (*pol* gene, motifs named RT, RH and IN), and *env* (envelope gene, motifs named SU and TM). Each colored box indicates one of the four parts: *gag* (red), *pro* (blue), *pol* (green), and *env* (orange). The features of the full-length chimpanzee-specific ERV insertions are described in the bottom of the figure.

**Figure 4:**
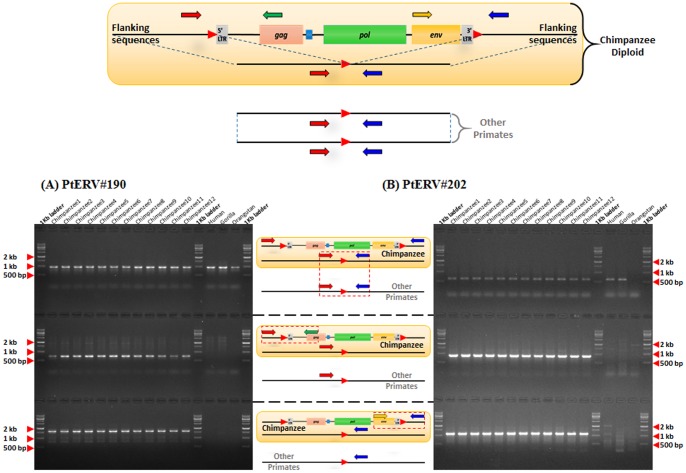
Polymorphic rcPtERVs in chimpanzee individuals. Two of eight potential rcPtERV loci were polymorphic and the PCR results are shown in this figure. In the strategy for PCR amplification, the colored arrows indicate the positions of PCR primers; the red and blue arrows indicate forward and reverse flanking primers, respectively. Green and yellow arrows indicate reverse and forward internal primers which are specific to PtERV elements. PCR band in the upper gel picture indicates the absence of the rcPtERV while PCR band in the middle and bottom gel picture indicates the presence of the rcPtERV.

Structurally or functionally intact ERVs contain four genes (*gag*, *pro*, *pol*, and *env*). However, most ERVs do not have the capability to encode gene products due to substitutions or frame-shift mutations [25]. Thus, the majority of ERVs ultimately become non-functional proviruses over the course of their residing time. In addition, the homologous recombination between two LTRs can delete retrotransposition-competent ERVs, leading to solitary LTRs [26]. The recombination results from a high degree of sequence identity between two LTRs. To identify full-length chimpanzee-specific ERV copies with intact open reading frames (ORFs), we first used an ORF finder [27] and then confirmed the result using the RetroTector 10 program [28]. Through this combined strategy, we were able to detect nine potential rcPtERVs which were full-length, containing intact *gag*, *pro*/*pol*, and *env* genes. However, one potential rcPtERV element contained short poly (N) stretch regions. We sequenced the poly (N) stretch regions and excluded the potential rcPtERV from our data because it contained nonsense mutations in *pol* gene (accession number KJ535124). Thus, at least eight chimpanzee-specific ERV copies retaining intact ORFs (Figure 3) exist in the chimpanzee genome and they have the potential to retrotranspose or reinfect in the chimpanzee genome. Among them, three elements (PtERV#190, #202, and #206) belonged to CERV1 family and the others (PtERV#1, #14, #65, #256, and #257) belonged to CERV2 family. Thus, they have a potential to act as a viral pathogen or LTR-retrotransposon causing structural variations in the chimpanzee genome. The number of potential rcPtERVs is much higher than that of human-specific rcHERV-Ks: only three HERV-K elements were reported to have intact ORFs [12]. Furthermore, we estimated the polymorphism rate of the eight potential rcPtERVs using a common chimpanzee population panel. Two of the rcPtERVs (25%) were polymorphic in the chimpanzee panel (Figure 4). Both of them were CERV1 family and contained intact internal genes including *env*. This result suggests that the CERV1 family has maintained retrotranspositional activity over the extended period of chimpanzee evolution. A similar case with another retroelement, *Alu* element, was previously reported; relatively old *Alu* subfamily (∼20 myrs) showed its recent mobility in the human genome after the divergence of humans and chimpanzees [29].

**Figure 5:**
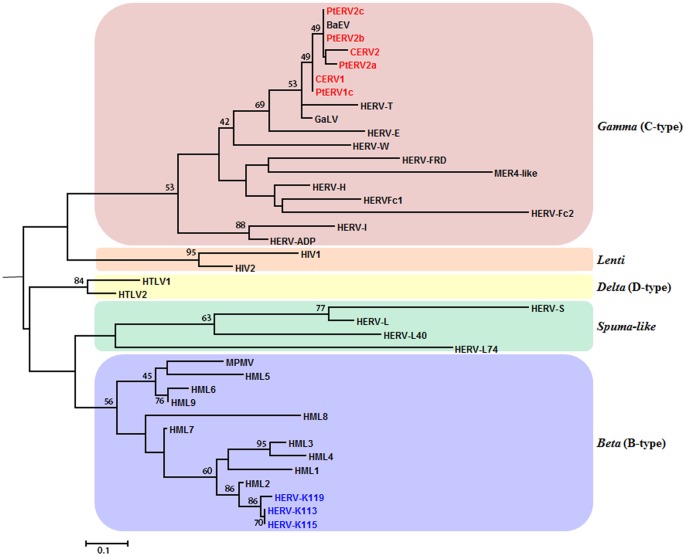
Phylogenetic relationship of different retroviral genera. Maximum-likelihood (ML) dendrograms (1000 bootstraps consensus) of five primate specific-retroviral genera (beta-, gamma-, delta-, lenti-, and spuma-like retrovirus), which are constructed based on the sequences of their Pol reverse transcriptase (RT) and integrase (IN). Chimpanzee-specific ERV copies and HERV-Ks are written in red and blue, respectively. The black scale bar denotes a genetic distance of 0.1 amino acid substitutions per position.

There are seven different retroviral genera: alpha-, beta-, gamma-, delta-, epsilon-, lenti-, and spuma-like retroviruses [30]. To investigate the sequence identity levels of PtERV families among the retroviral genera, we constructed a phylogenetic tree based on the amino acid sequence of the *pol* genes from the chimpanzee-specific ERV copies and the retroviruses above. Alpha- and epsilon-like retroviruses were eliminated from this phylogenetic analysis because they cause lots of background noise in sequence alignment. Similar to the results from RetroTactor 10, the two PtERV families, CERV1 and CERV2, were most closely related to the Baboon endogenous virus belonging to the genus of gamma retrovirus (Figure 5). This result agrees with the result from a previous study [1]. Based on the comparison of CERV1 and CERV2 families based on their copy numbers and the proportion of intact ERV elements, we found that the CERV1 family is older than the CERV2 family. To support this finding, we estimated the mutation frequencies of the chimpanzee-specific ERV subfamilies because generally, the element with the higher mutation frequency is the older element. Using the MEGA 5.1 program with pairwise distance, we aligned 5′ and 3′ LTR sequences from all members of each PtERV family. As shown in Table 3, the result also supports that CERV1 emerges earlier than CERV2 in the chimpanzee genome: the mutation frequency of the CREV1 family was 4% and that of CERV2 family was 1.1%. To estimate the age of the chimpanzee-specific ERV subfamilies, we performed the NETWORK analysis [31], based on the divergence among all the LTR sequences of each subfamily. As shown in Table 3, the age of CERV1 family are estimated to be in the range of 8.80–11.45 myrs old and are older than that of CERV2 family. It suggests that CERV1 family accumulated mutations more than CERV2 family after their insertions [27], [32]. Therefore, these results hold a potential explanation to the observed result that most potential rcPtERVs belong to the CERV2 family, although the majority of chimpanzee-specific ERV copies belong to the CERV1 family.

**Table 3:** Age estimate of PtERV subfamilies.

Subfamily	No. of LTRs aligned	Average length (bp)	Analyzed length (bp)	Pairwise distances without INDEL	Age (myrs)^a^
CERV1	80	409	345	0.037	8.32–10.82
PtERV1c	104	379	311	0.044	9.29–12.07
CERV2	2	544	532	0.009	1.70–2.21
PtERV2a	4	586	573	0.003	0.88–1.14
PtERV2b	2	491	474	0.004	0.80–1.04
PtERV2c	10	624	578	0.029	6.33–8.23

a The age is calibrated by 0.26% [48] and 0.20% [49] per site per myr (see Methods).

### Non-classical PtERV insertions (NCPIs)

The typical chimpanzee-specific ERV copies produced via LTR retrotransposition are flanked by short TSDs (2–5 bp). Thus, TSD is a hallmark for classical LTR-mediated insertion. Through TSD analysis of chimpanzee-specific ERV copies, we identified seven non-classical PtERV insertions (NCPIs) from our data. Among the seven NCPIs, four and three events were chimpanzee-specific solitary LTR copies and full-length chimpanzee-specific ERV copies, respectively. Non-classical insertions typically lack TSD and accompany genomic deletions (Table S6). Deletion happens not after chimpanzee-specific ERV insertion but either before chimpanzee-specific ERV insertion or at the same time as the insertion because it is very unlikely that any deletion event deletes only upstream and downstream sequences of chimpanzee-specific ERV, leaving the element intact. The mechanism responsible for the non-classical insertion of retroelements is not fully understood, but it has been suggested that a non-classical insertion is the remnant of a double strand break (DSB) repair. When DSB occurs at a pre-insertion site of chimpanzee-specific ERV, imprecise non-homologous end joining (NHEJ) could lead to integration of partial chimpanzee-specific ERV sequences into the genomic region [33]–[36]. To test this hypothesis, we investigated the breakpoint junction between a chimpanzee-specific ERV and its flanking sequence to identify microhomology sequences, which may mediate the formation of a bridge between the chimpanzee-specific ERV and its pre-insertion site (Figure S4). As shown in Table S6, five out of seven NCPIs have 2 or 3 bp of microhomology at their target insertion sites. In fact, NHEJ's repairing of DSB is one of major mechanisms for maintaining the host's genomic stability [37]–[40]. However, we could not rule out other mechanisms that may be responsible for NCPI.

We tried to estimate the size of the deletions associated with the NCPI by triple - alignment of human, chimpanzee, and orangutan genome sequences. The result showed that the seven NCPIs have deleted a total of 51,366 bp of genomic sequences from the chimpanzee genome since the divergence of humans and chimpanzees (Table 4); the size of the largest deletion was 37 kb. To validate the authenticity of NCPIs, we tried to amplify them through PCR and were successful in amplifying all of them. In addition, we examined the polymorphism level of the NCPIs using the chimpanzee population panel above, and found that all of them were fixed for the panel. Based on their environmental characteristics and polymorphic levels, we believe that, similar to other classical PtERV insertions, the NCPIs have also undergone natural selection.

**Table 4:** Non-classical transposable element insertions in the chimpanzee genome.

Chimpanzee	NCPIs	ARDs^a^	L1IMDs^b^
Number of event	7	14	26
Deletion size	51366 (bp)	466 (bp)	14923 (bp)
Mean of deletion size	7338 (bp)	33 (bp)	574 (bp)
Range of deletion size	37200 (bp)	204 (bp)	2965 (bp)

a *Alu* retrotranspositon-mediated deletion [37].

b LINE-1 insertion-mediated deletion [39].

### Chimpanzee-specific ERV causing alternative transcripts

**Figure 6:**
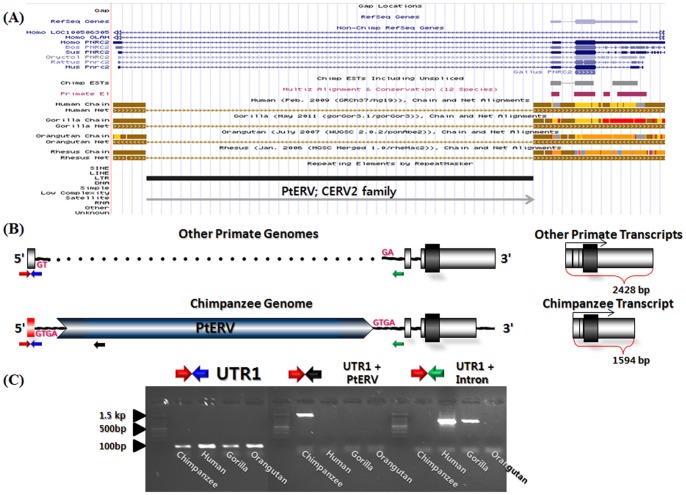
Alternative *PNRC2* transcripts caused by a chimpanzee-specific ERV. (A) Using the UCSC Genome Browser, the *PNRC2* gene is anatomized into intron, un-translated region (UTR), and coding exon. (B) The structures of chimpanzee and non-chimpanzee primate *PNRC2*s and their transcripts are depicted. The black and gray boxes indicate an exon and UTRs, respectively, and the red box denotes the untranscribed UTR1 of the chimpanzee *PNRC2*. Four different primers used to amplify PCR products are shown in red, blue, green, and black arrows, respectively. The target site duplications (TSDs) of the full-length chimpanzee-specific ERV are written in red. (C) The picture shows an agarose gel image of PCR products. Genomic DNAs from four different primates (human, chimpanzee, gorilla, and orangutan) were used as a DNA template for each PCR reaction.

The expression levels of chimpanzee sperm development-associated genes were critically modified by various mechanisms including alternative splicing [15], [16]. We detected one interesting locus (PtERV#1) from our data; it created an alternative transcript of a chimpanzee gene. Proline-rich nuclear receptor co-activator 2 (*PNRC*2; NM_017761.3) gene is located on the chimpanzee chromosome 1p36 (chr1:23,992,206-23,994,104). Human *PNRC*2 has a short ORF encoding 139 amino acids, and this gene contains 5′ untranslated regions (UTR) 1 and 2, which are separated by an intron. However, using *in silico* analysis, we found that the chimpanzee *PNRC2* transcript (NM_001252002) is deficient of 5′ UTR1 but its correspondent transcripts from other primates including human (NM_017761.3) and rhesus macaque (NM_001287331) contain the 5′ UTR1. We suggest that the chimpanzee-specific ERV residing on the genomic region between chimpanzee *PNRC2* 5′ UTR1 and 2 could induce an alternative splicing or a different RNA polymerase II binding site on the gene (Figure 6). Therefore, the chimpanzee-specific ERV can create alternative transcripts through its novel insertion within a gene.

## Conclusion

A previous study about PtERVs has identified 42 different PtERV families in the chimpanzee genome, using BAC read-pair analysis and genotyping via southern hybridization. Among them, only two families are chimpanzee-specific. Thanks to advanced genome sequencing technology, the whole chimpanzee genome sequence is currently available. Using the chimpanzee reference genome sequences, we identified 256 chimpanzee-specific ERV insertions, which cover most of the previously identified chimpanzee-specific ERV copies. In this study, we found eight PtERVs which are potentially retrotransposition-competent. Under a sound genomic condition, they could produce their progenies into new genomic regions in the chimpanzee genome. Depending on where PtERVs are integrated, they have a potential to disrupt a gene and change the gene expression level. In fact, one (i.e., PtERV#1) of the potential rcPtERVs has integrated within a chimpanzee gene and could be related to chimpanzee-specific transcript variants. We identified seven NCPI events and found that all of them are associated with genomic deletions of various sizes in the chimpanzee genome. Taken together, our study suggests that, since the divergence of chimpanzees and humans, PtERVs have contributed to species-specific genomic changes in the chimpanzee genome and could be a source which has been causing genomic differences between the chimpanzee and its closest relative, the human.

## Materials and Methods

### Data mining and manual inspection for chimpanzee-specific ERV loci

To identify chimpanzee-specific ERV loci, we first extracted all PtERV loci from the chimpanzee reference genome sequence (PanTro3; October. 2010 freeze) by using UCSC Table Browser utility [41]. In UCSC Table Browser, group and track were set as “Repeats” and “RepeatMasker”, respectively. Next, repName was assigned to “PtERV” in the filter section. The PtERV consensus sequences were shown in supplementary data. Then, we extracted 2 kb of flanking sequence on either side of each extracted PtERV and used the flanking sequence as a query for BLAST-Like Alignment Tool (BLAT) search against human (hg19; February 2009 freeze), gorilla (gorGor3; May 2011 freeze), and orangutan (ponAbe2; July 2007 freeze) reference genome sequences. In case where the query matches the three non-chimpanzee primate genomes, we retrieved the respective sequences and scrutinize repetitive elements residing the sequences by using CENSOR software tool [42] and RepeatMasker utility [43] When none of the three sequences contains the correspondent element to the PtERV, we considered the PtERV as a chimpanzee-specific ERV.

To study the structure of full-length chimpanzee-specific ERV copies, we used the ORF finder [44] and RetroTector 10 program [28].

### DNA preparation and PCR amplification

We conducted PCR assay to verify the authenticity of the chimpanzee-specific ERV. Four different genomic DNAs from *Pan troglodytes* (common chimpanzee), *Homo sapiens* (human; NA10851, Coriell Cell Repository, Camden, NJ), *Gorilla gorilla* (gorilla), and *Pongo pygmaeus* (Bornean orangutan) were used as a DNA template for each PCR reaction. The genomic DNAs from three apes and 12 related chimpanzee individuals were kindly provided by Dr. Takenaka (Primate Research Institute, Kyoto University). We designed oligonucleotide primers for each PCR reaction by using the software Primer3 [45] (Table S3 and S4) and PCR amplification of each chimpanzee-specific ERV was performed in 25 µL reaction using 10-20 ng DNA, 200 nM of each oligonucleotide primer, and 10 µL of PCR master mix containing DNA polymerase (TaKaRa EmeraldAmp GT PCR Master Mix, TaKaRa, Japan). Each PCR amplification was started with an denaturation step of 5 min at 95°C, followed by 35 cycles of 30 sec of denaturation at 95°C, 30 sec of annealing temperature, and 1 to 3 min of extension (depending on the expected size of PCR product) at 72°C, followed by a final extension at 72°C for 5 min. Two microliters of the resulting PCR products were loaded on 1% agarose gel, stained with ethidium bromide, and visualized using UV fluorescence. In case where the expected size of PCR product is longer than 2 kb, we alternatively used Ex TaqTM polymerase (TaKaRa Japan), 2X EF-Taq DNA polymerase (SolGent, Korea), or KOD FX (Toyobo, Japan).

### Multiple sequence alignments and phylogenetic analysis

We downloaded the consensus sequences of six PtERV subfamilies from Genetic Information Research Institute [42] and aligned chimpanzee-specific ERV copies with these consensus sequences by using the software BioEdit v.7.0.5.3 [46]. To construct a maximum likelihood (ML) phylogenetic tree among the chimpanzee-specific ERV copies, we used the Molecular Evolutionary Genetics Analysis (MEGA) software 5.1 with a Kimura-2-parameter distance [47]. To investigate the taxonomic relationship between chimpanzee-specific ERV subfamilies and the five retroviral genera [1], [30], we also constructed a maximum-likelihood (ML) phylogenetic tree, based on their Pol amino acid sequence, by using MEGA 5.1 with Jones-Taylor-Thornton (JTT) model. The support for each node of the tree was evaluated based on 1000 bootstrap replicates. The ages of the chimpanzee-specific ERV families were estimated with NETWORK 4.611 [31]. Given that LTR sequences accumulate mutations at the neutral rate after the integration, we used and calibrated two different mutation rates, 0.26 [48] and 0.20% [49] per site per myr, for the age estimation.

## Supporting Information

Figure S1 Sequence variants in CERV1 and CERV2 families. We retrieved 5′ and 3′ LTR consensus sequences of each subfamily of CERV1 and CERV2 families and aligned them using software BioEdit v.7.0.5.3. (a) CERV1 and PtERV1c LTR sequences are showed in purple and light-blue, respectively. The colored boxes on the aligned sequences denote nucleotide positions of the sequence variation between different subfamilies of CERV1. (b) CERV2, PtERV2a, PtERV2b, and PtERV2c LTR sequences are showed in red, yellow, green, and blue, respectively. The colored boxes on the aligned sequences denote nucleotide positions of the sequence variation between the different subfamilies of the CERV2 families.
(PDF)

Figure S2 Maximum likelihood tree of chimpanzee-specific ERV copies. For each PtERV subfamily, we aligned its LTR consensus sequences and the LTR sequences from all of its members: A, CERV1. B, PtERV1c. C, CERV2, PtERV2a, and PtERV2b. D, PtERV2c. The consensus sequences are denoted by red letters. The scale bars indicate an evolutionary distance.
(PDF)

Figure S3 Polymorphic pattern of chimpanzee-specific ERV copies. The PCR amplification of each chimpanzee-specific ERV locus was performed with a common chimpanzee population panel composed of 12 related individuals and three other primate samples. (A) In the strategy for PCR amplification, the green, yellow, and red chevrons represent internal region, LTRs, and TSDs, respectively. The red and blue arrows indicate forward and reverse primers, respectively. Green and yellow arrows indicate internal primers which are specific to PtERV element. (B) Gel chromatography of PCR products exhibits all of the three possible alleles of a full-length chimpanzee-specific ERV locus. (C) Fixed and polymorphic solitary-LTR loci.
(PPTX)

Figure S4 PCR amplification and multiple sequence alignment of NCPI events. (A) The PCR amplification of representative NCPI locus (PtERV#33) (B) In the multiple sequence alignment of PtERV#33 with other primate sequences, the red and blue boxes indicate deleted sequences and microhomology sequences (TAA), respectively.
(PPTX)

Table S1 Summary of full-length chimpanzee-specific ERV copies.
(XLSX)

Table S2 Summary of chimpanzee-specific solitary LTR copies.
(XLSX)

Table S3 Primer information for the PCR amplification of full-length chimpanzee-specific ERV copies.
(XLSX)

Table S4 Primer information for the PCR amplification of chimpanzee-specific solitary LTR copies.
(XLSX)

Table S5 Polymorphism pattern of the 11 polymorphic chimpanzee-specific ERV copies.
(XLSX)

Table S6 NCPI events in the chimpanzee genome.
(XLSX)

Data S1 Consensus sequences of PtERV subfamilies from Repbase [42].
(TXT)
