# Supplementary material for: Chimpanzee-Specific Endogenous Retrovirus Generates Genomic Variations in the Chimpanzee Genome
Source: PLoS One. 2014 Jul 2;9(7):e101195. doi: 10.1371/journal.pone.0101195 (PMC4079660; doi:10.1371/journal.pone.0101195)
Supplement: Figure S1 — Sequence variants in CERV1 and CERV2 families. We retrieved 5′ and 3′ LTR consensus sequences of each subfamily of CERV1 and CERV2 families and aligned them using software BioEdit v.7.0.5.3. (a) CERV1 and PtERV1c LTR sequences are showed in purple and light-blue, respectively. The colored boxes on the aligned sequences denote nucleotide positions of the sequence variation between different subfamilies of CERV1. (b) CERV2, PtERV2a, PtERV2b, and PtERV2c LTR sequences are showed in red, yellow, green, and blue, respectively. The colored boxes on the aligned sequences denote nucleotide positions of the sequence variation between the different subfamilies of the CERV2 families. (PDF) [file pone.0101195.s001.pdf]

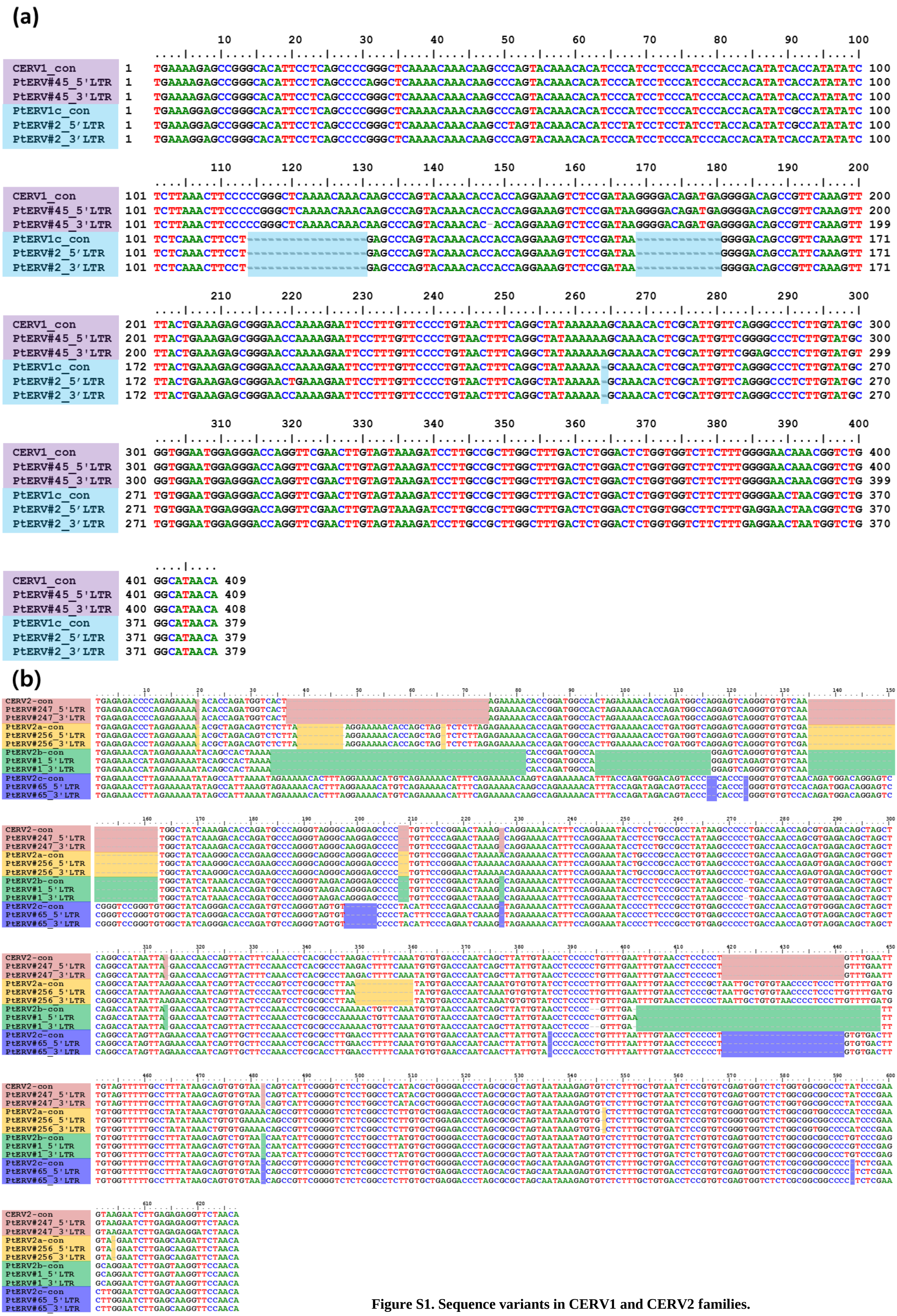

Figure S1. Sequence variants in CERV1 and CERV2 families.
