# Supplementary figures and images for: Chimpanzee-Specific Endogenous Retrovirus Generates Genomic Variations in the Chimpanzee Genome
Source: PLoS One. 2014 Jul 2;9(7):e101195. doi: 10.1371/journal.pone.0101195 (PMC4079660; doi:10.1371/journal.pone.0101195)

(a)

PtERV#33

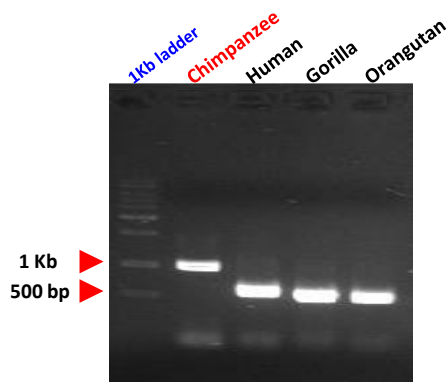

(b)

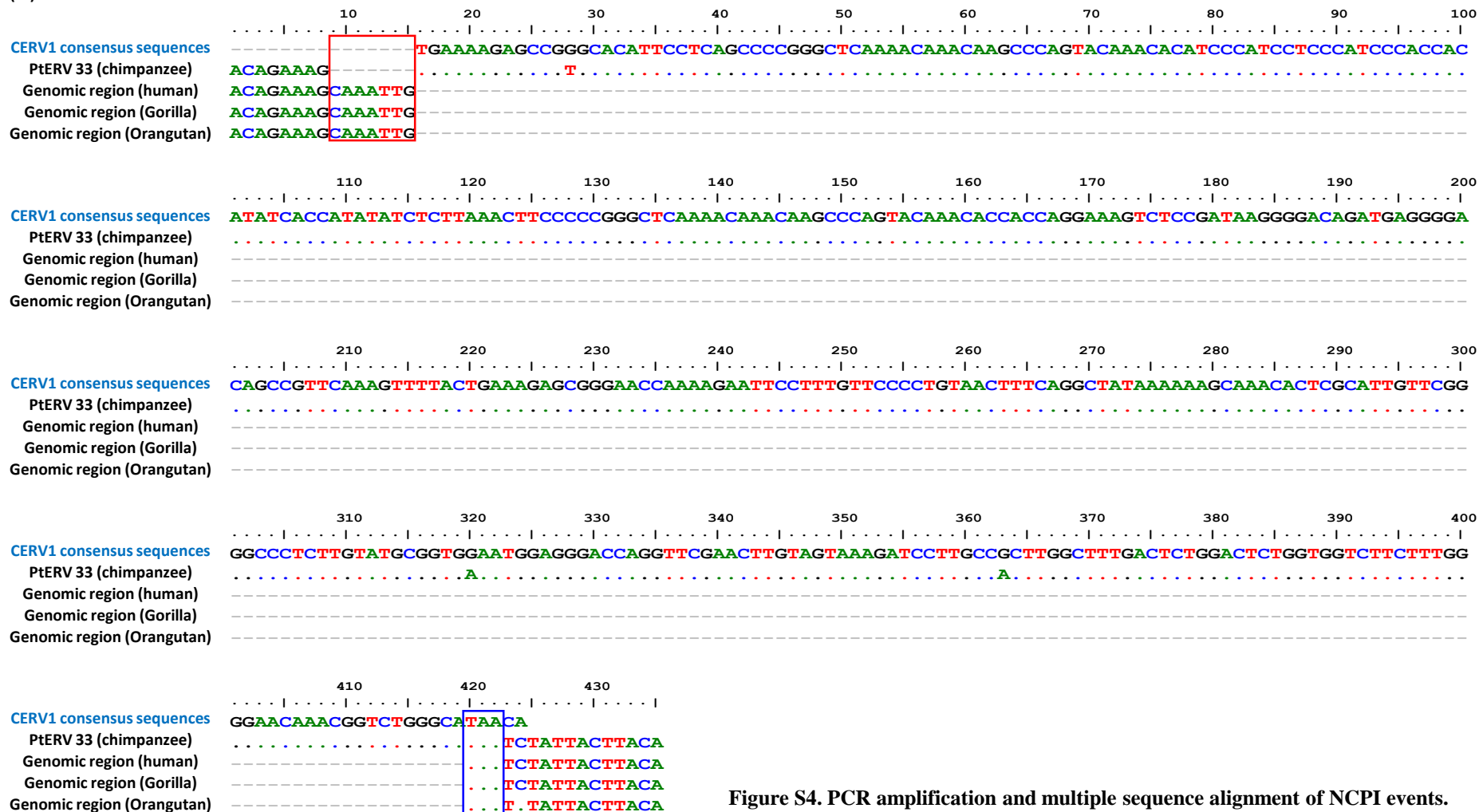

Figure S4. PCR amplification and multiple sequence alignment of NCPI events.

Supplement: Figure S4 — PCR amplification and multiple sequence alignment of NCPI events. (A) The PCR amplification of representative NCPI locus (PtERV#33) (B) In the multiple sequence alignment of PtERV#33 with other primate sequences, the red and blue boxes indicate deleted sequences and microhomology sequences (TAA), respectively. (PPTX) [file pone.0101195.s004.ppt]
