# Supplementary material for: Chimpanzee-Specific Endogenous Retrovirus Generates Genomic Variations in the Chimpanzee Genome
Source: PLoS One. 2014 Jul 2;9(7):e101195. doi: 10.1371/journal.pone.0101195 (PMC4079660; doi:10.1371/journal.pone.0101195)
Supplement: Figure S2 — Maximum likelihood tree of chimpanzee-specific ERV copies. For each PtERV subfamily, we aligned its LTR consensus sequences and the LTR sequences from all of its members: A, CERV1. B, PtERV1c. C, CERV2, PtERV2a, and PtERV2b. D, PtERV2c. The consensus sequences are denoted by red letters. The scale bars indicate an evolutionary distance. (PDF) [file pone.0101195.s002.pdf]

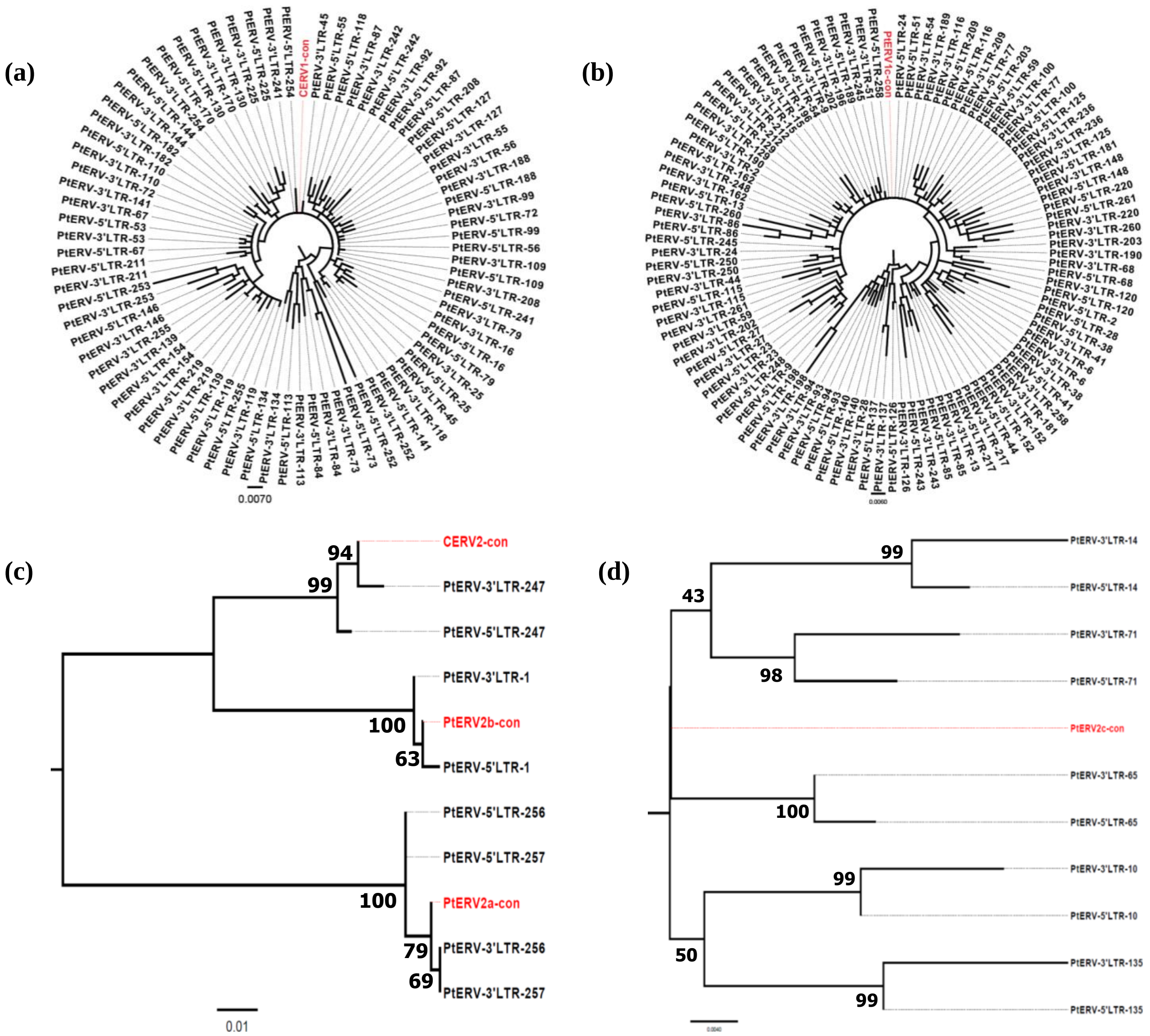

Figure S2. Maximum likelihood tree of chimpanzee-specific ERV LTR copies.
